# Primary Hepatic Angiosarcoma Presenting As Cryptogenic Cirrhosis

**DOI:** 10.7759/cureus.43529

**Published:** 2023-08-15

**Authors:** Ricardo Anguiano-Albarran, Daniel Cain, Franklin Obi, Sidart Pradeep, Michael Cimo, Shivang Mehta

**Affiliations:** 1 Internal Medicine, Baylor Scott & White All Saints Medical Center Texas Christian University (TCU) - Internal Medicine Residency, Fort Worth, USA; 2 Pathology, Baylor Scott & White All Saints Medical Center, Fort Worth, USA; 3 Transplant Hepatology, Baylor Scott & White All Saints Medical Center, Fort Worth, USA; 4 Transplant Hepatology, Annette C. & Harold C. Simmons Transplant Institute, Fort Worth, USA

**Keywords:** gastroenterology, hepatology, cirrhosis, cryptogenic cirrhosis, hepatic angiosarcoma

## Abstract

Primary hepatic angiosarcoma (PHA) is an exceedingly rare and aggressive neoplasm of mesenchymal origin. PHA makes a very small portion of primary liver tumors and conveys a poor prognosis. Symptomatology can be vague and often mimics primary hepatocellular carcinoma upon presentation. Diagnosis requires careful immunohistopathologic confirmation. We present a case of PHA in a patient with abdominal pain and suspected underlying cryptogenic cirrhosis.

## Introduction

Angiosarcomas are malignant mesenchymal neoplasms arising from lymphatic or blood endothelial cells. In general, angiosarcomas are associated with high mortality. In the liver, PHA induces aggressive normal endothelial cell replacement, which triggers hepatocyte atrophy, sinusoidal obliteration, and the formation of complex vascular bed networks. Patients usually present in their sixth decade of life, with a male-to-female ratio of 3-4:1 [1]. PHA accounts for less than 1% of all primary malignant liver tumors and roughly 2% of all soft tissue sarcomas [2,3]. It is estimated that in the United States, the annual incidence is about 0.14 to 0.25 cases per million people [4].

Risk factors for the development of PHA include vinyl chloride, chronic arsenic ingestion, androgenic anabolic steroids, oral contraceptives, phenelzine, hemochromatosis, and von Recklinghausen disease [1,2,5,6]. Exposure to thorium oxide and ionizing radiation has also been proposed as a risk factor, with thorium no longer being used in regular clinical practice [7,8].

Clinical presentation can be vague, with non-specific symptoms including abdominal pain, abdominal distention, fever, anemia, and unintentional weight loss [2,9]. In a subset of instances, an acute life-threatening hemorrhagic shock presentation has been reported [10,11]. In addition to the complications of tumor rupture, coagulopathies such as disseminated intravascular coagulation and the Kasabach-Merritt phenomenon have been associated with PHA [12,13]. Liver function is often intact until the later stages of the disease [14]. To date, no therapeutic intervention has been successful. An orthotopic liver transplant has been attempted, but the longest reported survival was 10 months post-transplantation [15].

## Case presentation

A 72-year-old male presented to our facility with dull right upper quadrant pain and new onset jaundice over a two-week period. He had a history of obesity (BMI 48.29 kg/m²), uncontrolled diabetes mellitus, and pulmonary embolism on anticoagulation. Associated symptoms included generalized fatigue, dyspnea on exertion, and decreased appetite. The patient also reported unintentional weight loss over the course of the last year. He denied a history of heavy alcohol consumption or known chronic liver disease. He had no known exposure to ionizing radiation, arsenic, or vinyl chloride.

Upon initial evaluation, the patient was hemodynamically stable with notable edema in the lower extremities, spider nevi, and abdominal distension with tenderness to palpation along the right abdominal quadrants. There was no prominent ascites, and the patient did not display neurologic symptoms suggestive of encephalopathy. There was no obvious hepatosplenomegaly.

Initial laboratory results (Table 1) were significant for apparent coagulopathy, hepatic dysfunction, and renal injury. Platelet count decreased with an elevated international normalized ratio (INR) of 1.7. Prothrombin and activated partial thromboplastin times were within the normal range. Hemoglobin was mildly anemic initially with a significant decrease during the hospital course. Blood urea nitrogen (BUN) and serum creatinine were both elevated. Hepatic chemistries demonstrated an elevated total bilirubin level, alkaline phosphatase (ALP), aspartate aminotransferase (AST), alanine aminotransferase (ALT), and low albumin. The model for end-stage liver disease-Na score was 32.

**Table 1:** Lab Values From Admission to Discharge

Lab Study	Results (Admission->Discharge)	Reference Range
Total Bilirubin	3.9->9.6mg/dL	0.2-1.0 mg/dL
Direct Bilirubin	6.6mg/dL	0.0-0.2mg/dL
Alkaline Phosphatase (ALP)	152->99 U/L	45-177 U/L
AST (SGOT)	192->246 U/L	15-37 U/L
ALT (SGPT)	84->95 U/L	16-61 U/L
Total Protein	5.8->4.6 g/dL	6.4-8.2 g/dL
Albumin	2.3->2.4 g/dL	3.4 -5.0 g/dL
White Blood Cell (WBC)	7.1->7.1 K/uL	4.5-11.0 K/uL
Hemoglobin	13.8->8.6 g/dL	13.5->18.0 g/dL
Hematocrit	41.0->26.4 %	40.0-52.0%
Platelets	29->20 K/uL	140-440 K/uL
Prothrombin Time (PT)	19.5->23.0s	9.4-12.5s
International Normalized Ratio (INR)	1.7->1.9	
Partial Thromboplastin Time (PTT)	40.8->43.8s	25.1-36.5s
Fibrinogen	318mg/dL	200-400 mg/dL
Iron	77 ug/dL	65-175 ug/dL
TIBC	169 ug/dL	250-450 ug/dL
Ferritin	1402 ng/dL	24-380 ng/mL
% Iron Saturation	46%	20-55%
Hepatitis A IgM Antibody	Non-Reactive	
Hepatitis B Surface Antigen	Non-Reactive	
Hepatitis B Core IgM Antibody	Non-Reactive	
Hepatitis C Antibody	Non-Reactive	
Antinuclear Antibody (ANA) Screen	Positive	Negative
ANA Pattern	Nucleolar	None
ANA Titer	1:.160	None
Anti-Smooth Muscle Antibody (ASMA)	Negative	Negative
Anti-Mitochondrial Antibody (AMA)	Negative	Negative
Alpha Fetoprotein (AFP)	1.1 ng/mL	<6.1 ng/mL
Carcinoembryonic Antigen (CEA)	1.2 ng/mL	0.0-2.4 ng/mL
CA 19-9	<3 U/mL	<34 U/mL

**Figure 1:**
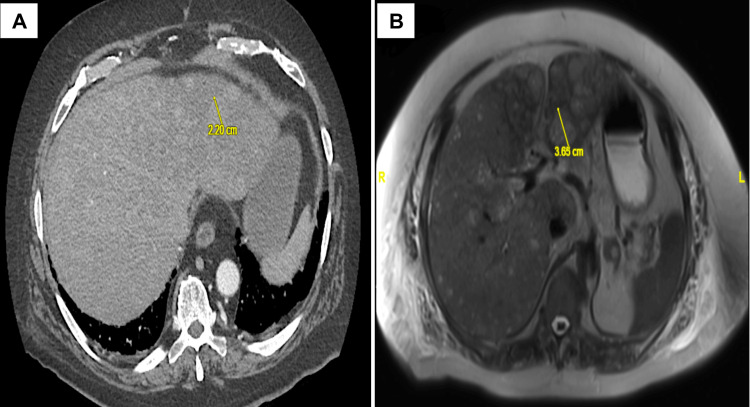
A: Transverse CT abdomen demonstrating a lesion in the left hepatic lobe. B: Follow-up MRI with diffuse lesions throughout the liver.

Computed tomography (CT) of the abdomen was then completed, which demonstrated a nodular liver contour concerning for cirrhosis (Figure 1A). There were numerous areas of nodular enhancement predominantly in the left hepatic lobe concerning for hepatocellular carcinoma (HCC). Magnetic resonance imaging (MRI) highlighted the masses with diffuse distribution throughout the liver (Figure 1B). These findings were concerning for metastatic disease versus primary advanced HCC.

**Figure 2:**
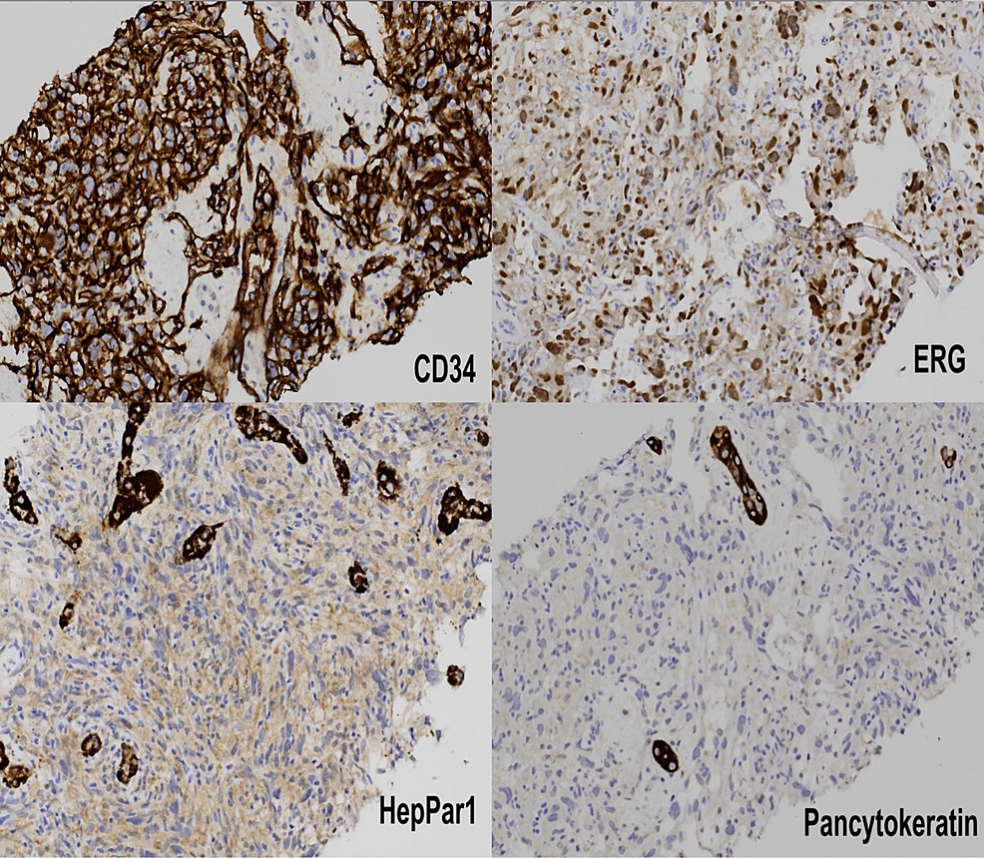
Special Histopathological Stain Slides of the Liver Mass Stains demonstrating cells with highly pleomorphic nuclei, dark chromatin, increased karyorrhexis, mitotic activity occasionally lining well-defined vascular spaces, and often intimately admixed with surrounding hepatocytes.

The serologic screening was unremarkable for viral hepatitis and autoimmune etiologies. Alpha-fetoprotein, carbohydrate antigen 19-9, and carcinoembryonic antigen tumor markers were all negative. Transjugular liver biopsy was pursued revealing a hepatic venous pressure gradient (HPVG) of 11 mmHg. Biopsy samples were sent for comprehensive immunohistochemical staining. Sample staining returned positive for CD34 and ETS family transcription factor ERG (ERG), confirming underlying PHA (Figure 2). In addition, pancytokeratin and anti-hepatocyte specific antigen (HepPar 1) stains were negative (Figure 2) ruling out HCC. Trichrome staining highlighted areas of fibrosis, some of which were pericellular. Iron stain and Periodic acid-Schiff (PAS) stain with diastase were negative. The elevated hepatic venous pressure gradient (HVPG) was suspected to be related to the level of malignancy seen after the biopsy.

Ultimately, no intervention was pursued, as the patient desired to transition to comfort care and was subsequently discharged to hospice.

## Discussion

There are no treatment guidelines for PHA. A preferred treatment option has been surgical resection with adjuvant therapy, but data regarding outcomes is scarce. Transplantation has been abandoned due to poor outcomes and high rates of recurrence [14]. Unfortunately, most cases of PHA are discovered in advanced stages, and very few of these patients are deemed candidates for surgical management. While PHA is a contraindication for a liver transplant due to poor post-transplant outcomes, transcatheter arterial chemoembolization (TACE) is a palliative option for patients with unresectable PHA.

Elevated hepatic chemistries (alanine transaminase (ALT), aspartate aminotransferase (AST), alkaline phosphatase (ALP)) along with thrombocytopenia and anemia is a common pattern in laboratory analysis [2]. Tumor markers common to gastrointestinal and hepatic malignancies are often within normal limits in these patients [2]. From a radiologic standpoint, PHAs demonstrate significant heterogeneity. CT imaging of these tumors can be either hypo or hyperdense, depending on the presence of intra-tumor hemorrhage. On T2-weighted MRI, dominant masses of PHA can have internal structural heterogeneity similar to HCC [16]. While imaging modalities can aid in the detection of suspicious hepatic lesions, the heterogeneity of PHA and variation in presentation can make lesions difficult to distinguish. Therefore, diagnosis requires immunohistochemical analysis.

A typical appearance on histological examination is a highly vascularized tumor with variable vascular patterns accompanied by spindle-shaped and polyhedral cells [17]. Immunohistology staining screens for ERG expression, CD31, CD34, and Factor VIII-Related Antigen (FVIIIRAg). ERG is a relatively newer biomarker included in the evaluation of PHA and has been proposed to be a more specific and consistent finding in hepatic angiosarcomas [18]. CD31 stains for a transmembrane glycoprotein active in angiogenesis. It is highly sensitive and specific for tumors of vascular origin [17,19]. Similarly, CD34 stains for a hematopoietic progenitor cell antigen that is prevalent in areas of active vascular differentiation [20]. The focal appearance of FVIIIRAg can be highly specific but less sensitive. This marker can be unreliable if the tumor is highly differentiated [20].

## Conclusions

In patients presenting with clinically apparent hepatic dysfunction and concomitant suspicious lesion(s) on imaging, PHA should be included in the differential. Lesions should be investigated with a multi-modal approach with the pursuit of a liver biopsy at the discretion of the multidisciplinary team. Early detection and localized tumor burden may provide the opportunity for surgical intervention. Our case emphasizes the need for a definitive pathologic evaluation and the rarity of PHA mimicking a primary HCC in the setting of cryptogenic cirrhosis.
